# Omics in mini-livestock: a genomic perspective on the future of sustainable food systems

**DOI:** 10.3389/fgene.2025.1740301

**Published:** 2026-01-08

**Authors:** Alexander Sode, Arindam Halder, Julia Metzger

**Affiliations:** Veterinary Functional Genomics, Institute of Animal Genomics, University of Veterinary Medicine Hannover, Hannover, Germany

**Keywords:** animal improvement, epigenomics, genomics, metabolomics, mini-livestock, omics, proteomics, sustainability

## Abstract

Mini-livestock refers to small vertebrates and invertebrates used as human food, animal feed, or for other beneficial purposes. They represent sustainable alternatives to conventional livestock, whose potential is now being revealed through advances in omics technologies. Omics approaches such as genomics, transcriptomics, proteomics, metabolomics, and epigenomics provide comprehensive insights into growth, reproduction, adaptation, and disease resistance of these species, enabling the identification of genetic markers to enhance breeding efficiency and to improve productivity. However, the application of omics technologies in mini-livestock remains limited due to challenges such as high costs, lack of reference genomes, and limited bioinformatics resources. Overcoming these barriers will be crucial for fully harnessing the potential of mini-livestock in improving global food security and environmental sustainability.

## 1 Introduction

“Mini-livestock,” from edible insects to small vertebrates, appear as a sustainable alternative to traditional livestock with hidden potential ready to be unleashed by the latest omics technologies. The term mini-livestock includes all vertebrates and invertebrates, which are used as human food, animal feed, a source of income, or for other applications for human benefit, and are at the same time extremely small forms of conventional farm animals or naturally small-scale mammals, birds, reptiles, molluscs or insects (Vietmeyer, 1984; National Research Council, 1991; Hardouin, 1995; Hardouin et al., 2003; Titilola et al., 2015; Ingweye and Kalio, 2020). Mini-livestock have a versatile use and were proposed to be highly beneficial in terms of raising them in small areas and clusters, especially in rural, peri-urban and urban regions to provide human food, capital or manure (Ogunjimi et al., 2012; Klapwijk et al., 2020). It is anticipated that, in the future, these animals will play an increasing role in food security and economically responsible food production (Branckaert, 1995; Paoletti and Dreon, 2005). Among other livestock, mini-livestock have been co-evolving over decades in stressful environments and adapted to harsh conditions with limited feed resources and water availability (Assan, 2013). As an example, edible mealworm species have an unpretentious nature, low water footprint and offer at the same time a protein-rich food resource (Miglietta et al., 2015; van Broekhoven et al., 2015; Sellem et al., 2024). In order to gain detailed insights into the biology of mini-livestock and to comprehensively understand the genetic basis of traits of interest for future breeding strategies, omics technologies such as genomics, epigenomics, transcriptomics, proteomics or metabolomics have been increasingly recognized as key tools for translating the genome into the phenome (Riggs et al., 2017; Rexroad et al., 2019; Yang X. et al., 2020; Chakraborty et al., 2022; Verardo et al., 2023). Particularly, DNA and RNA sequencing analyses have been frequently applied for conventional and mini-livestock, investigating specific characteristics such as body development and condition, feed conversion, meat quality, or growth (Hu et al., 2007; Lu et al., 2021; Posbergh and Huson, 2021; Manzanilla-Pech et al., 2022; Silva-Vignato et al., 2022; Zhang C. et al., 2022).

Therefore, the aim of this review is to give a comprehensive understanding of mini-livestock and their potential roles across various domains. It explores the application of different omics approaches in advancing mini-livestock research, particularly with regard to improving productivity, product quality, addressing biomedical questions and reducing greenhouse gas emissions and highlights existing limitations and challenges.

## 2 The concept of mini-livestock

The definition of mini-livestock has evolved over time, making the list of animals classified under this term broad, dynamic, and subject to change (Ingweye and Kalio, 2020). Prior to the current definition of mini-livestock, various earlier terms were employed to describe smaller farm animals (e.g., poultry, rabbits or guinea pigs), as well as breeds that are half of their original size, including micro-pigs or micro-cattle (National Research Council, 1991; Ingweye and Kalio, 2020). The term mini-livestock is not limited to endothermic animals, but also includes ectothermic species like snakes, snails, lizards, frogs, silkworms, honey bees and crickets (Cicogna, 1992; Defoliart, 1995; Hardouin, 1995; Imoru and Babadipe, 2019). Likewise, edible insects like the yellow mealworm, the common house fly or the black soldier fly were considered as mini-livestock and have the highest potential of being animal feed (Voulgari-Kokota et al., 2023).

In the mid-1980s in Latin America, researchers began to categorize livestock based on their different sizes, distinguishing between “main-frames” like cattle and “mini-frames” like sheep. Additionally, the term micro-livestock was introduced to describe small animals suited for household husbandry, especially in resource-poor urban or rural environments in the Global South (Vietmeyer, 1984; Titilola et al., 2015). Micro-livestock typically includes species that adapt to harsh environments, efficiently recycle nutrients, or utilize unconventional resources like rabbits, guinea pigs, or bees (Peters, 1987). However, the term “micro” has also been used more broadly to describe microorganisms, including yeast, fungi or bacteria that serve as a protein source (Hardouin, 1995).

Another early root of the term mini-livestock was the label “unconventional livestock”, referring to species that are not used in conventional agriculture or traditionally domesticated (Peters, 1987). To further differentiate such species, some authors proposed using a relative weight production index, which accounts for the live weight at purchase and meat yield of offspring in proportion to the annual availability of reproductive females (Cicogna, 2000). Based on this index, guinea pigs produced meat equivalent to 6–10 times their own live body weight per year, whereas cattle yielded only about 0.4 times their body weight annually (Cicogna, 2000).

Despite these different backgrounds, the use of the terms “unconventional livestock”, “micro-livestock” or “mini-livestock” remained inconsistent across scientific publications and other sources, which can lead to confusion regarding the species included by these concepts (Hardouin, 1995; Ingweye and Kalio, 2020). Mini-livestock were reported to reproduce quickly, in high numbers, and are economically efficient, resulting in a higher input-output ratio (Hardouin et al., 2003; Titilola et al., 2015). More recently, environmental sustainability has become a key criterion: mini-livestock often generate lower greenhouse gas emissions (CO2-equivalents) per unit of protein produced than conventional livestock (Schanes et al., 2016; Imoru and Babadipe, 2019; Ghosh et al., 2021; Bai et al., 2023). As livestock production is considered to be a large contributor to climate change, accounting for up to 14.5% of all anthropogenic greenhouse gas emissions, more and more efforts are made to study mini-livestock as a potential alternative protein resource (Gerber et al., 2013; Alexander et al., 2017). For example, a study on Global Warming Potential suggested that insect-based resources had a significant potential to reduce the carbon footprints of European consumers, especially when insects are directly consumed as food or used for feeding in broiler production systems (Van Alfen, 2014; Vauterin et al., 2021).

The emission intensity of greenhouse gas has been shown to vary widely across different countries, livestock species, breeds and production systems (Hyslop, 2008; Herrero et al., 2013). Among different strategies to mitigate emissions, breeding schemes that promote the selection of traits enhancing production efficiency, such as residual feed intake or longevity, were suggested to reduce overall emissions (Wall et al., 2010). Ruminants, in particular, served as a model species in this context, as they have been extensively studied for their potential to reduce enteric methane emissions through selective breeding (de Haas et al., 2021). Overall, these studies suggest that genetic selection, alongside improved management practices, offers substantial potential for reducing livestock emissions on a global scale. However, the growing impact of climate change highlights the limitations of mitigation-focused breeding approaches and the pressing need for breeding strategies towards climate-resilience to maintain system integrity (Mutale et al., 2025). Omics approaches present a critical resolution for the identification and selection of traits providing resilience to high temperatures, drought, and climate-driven diseases (Liu et al., 2015; Vitorino Carvalho et al., 2021; Reed et al., 2023; Shi et al., 2023; Feng X. et al., 2024; Karami et al., 2025). In addition to pure physiological tolerance, a variety of adaptive mechanisms have emerged from multi-omics analysis, such as microbially driven plasticity, nutritional stress-driven metabolic flexibility, and epigenetic control in response to varying environments (Wallberg et al., 2017; Guilliet et al., 2022; Sukmak et al., 2024). Furthermore, genomic understanding of photoperiodic and light responses opens a way to consolidate reproductive rhythms or ontogenetic trajectories affected by different climate regions (Morris et al., 2020; Yuyan et al., 2025; Zhao X. et al., 2025).

Building on this, the integration of multi-omics data into breeding programs is essential to unravel the complex biological mechanisms underlying trait variation, enabling more precise and effective selection strategies, not only in conventional livestock but also in emerging mini-livestock species, which have so far received limited attention in this regard (Berry et al., 2011; Yang et al., 2017; Fonseca et al., 2018; Ahmad et al., 2022; Liu and Penagaricano, 2025). While the primary focus of mini-livestock research is on species that contribute to sustainable food and feed production, it is important to recognize that breeding strategies for certain miniature vertebrates, such as laboratory-bred miniature pigs, have followed a different trajectory. These animals are not typically part of food-oriented mini-livestock systems but have made significant contributions as biomedical models, addressing important clinical and translational research questions (Arora et al., 2022; Chakraborty et al., 2022; Jia Y. et al., 2024; Miao et al., 2024b; Zhang J. et al., 2025).

## 3 A multi-omics perspective on mini-livestock

**FIGURE 1:**
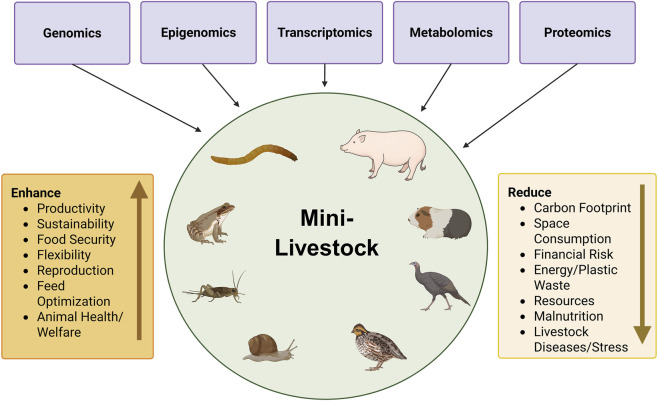
Integration of multi-omics approaches in mini-livestock research and their impacts. This figure illustrates how various omics technologies contribute to mini-livestock research. Created in BioRender, agreement no. PZ296612JU.

To fully exploit the genetic potential of mini-livestock in sustainable production systems (Figure 1), comprehensive multi-omics approaches, encompassing genomics, transcriptomics, proteomics, epigenomics, and/or metabolomics, are needed to decode the molecular basis of key traits (Riggs et al., 2017; Yang et al., 2017; Poma et al., 2022). The advent of omics technologies has transformed biological research by shifting the focus from isolated molecular components to the comprehensive analysis of complex biological systems under varying conditions (Hasin et al., 2017; Karczewski and Snyder, 2018). These technologies offer powerful tools for advancing mini-livestock research, enabling deeper insights into the molecular mechanisms underlying key traits (Poma et al., 2022; Xiong et al., 2023; Jia X. et al., 2024). Traditional breeding approaches, initially based on phenotypic selection and later supported by marker-assisted selection (MAS) based on Quantitative Trait Loci (QTL), have contributed significantly to genetic improvement (Meuwissen et al., 2001; Ikeobi et al., 2002; de Koning, 2016). However, these approaches faced limitations, particularly in identifying the genetic basis of complex traits due to high costs and limited resolution (Lande and Thompson, 1990; Grisel, 2000). The integration of genome-wide approaches, such as genome-wide association studies (GWAS), has further advanced our understanding of trait architecture and genetic variation across species, though such studies may still fail to capture systematically gene-gene and gene-environment interactions (Andersson, 2009; Ober et al., 2012; Fang et al., 2019). Subsequently, whole genome sequencing (WGS) has significantly improved the prediction accuracy compared to standard SNP arrays and allowed a more comprehensive assessment of genetic diversity across populations (Moghaddar et al., 2019; Xia et al., 2021). Further insights into the gene expression regulation have been offered by transcriptomics, highlighting the underlying mechanisms and gene-regulatory pathways for traits of interest (Xue Q. et al., 2017; Li J. et al., 2022). Beyond these studies solely focusing on the genetic code itself, epigenomics has emerged as a more recent field, which targets DNA modifications that affect gene expression without altering the DNA sequence itself (Wang and Ibeagha-Awemu, 2020; Zhou et al., 2020). Epigenetic processes, including DNA methylation, chromatin remodelling, histone modifications and non-coding RNA-activity, can lead to heritable changes in gene expression and have been shown to play key roles in development, adaptation, and phenotype variability (Lyko et al., 2010; Felsenfeld, 2014; Herman et al., 2014; Sarg et al., 2015). To fully understand how these regulatory mechanisms shape biological functions, proteomics adds another layer of information targeting the actual protein products and their roles in cellular processes (Haider and Pal, 2013; Aslam et al., 2017). Proteomic analyses enable the investigation of changes in protein levels, post-transcriptional modifications, and interactions in response to various stimuli (Franco et al., 2015; Sierra et al., 2021). Furthermore, metabolomics, as the downstream layer of omics, focuses on the comprehensive profiling of small-molecule metabolites. By examining the metabolome, researchers can gain insights into metabolic pathways and how they impact cellular processes and phenotypic variations (Poma et al., 2022; Du et al., 2025). Complementing these host-centric approaches, metagenomics analyses the collective genomes of microbiota, revealing how microbial communities influence digestion, immune competence, and environmental resilience as factors especially important to the productivity and sustainability of mini-livestock species (Yang et al., 2022; Hou et al., 2024).

To fully exploit the complementary nature of these omics’ layers, integrative analytical frameworks are increasingly required. Multi-omics integration enables the joint analysis of diverse molecular layers to understand biological mechanisms that are not detectable when studied in isolation (Hasin et al., 2017; Baiao et al., 2025). Classical approaches rely on correlation-based techniques such as Canonical Correlation Analysis (CCA), which identifies shared patterns across datasets and has been widely applied to multi-omics applications (Jiang et al., 2023). Network-based strategies, including Weighted Gene Co-expression Network Analysis (WGCNA), enable the construction of cross-layer regulatory modules and facilitate the identification of key molecular drivers underlying complex traits (Langfelder and Horvath, 2008). Complementary knowledge-driven approaches, such as pathway and functional enrichment analysis, integrate multi-omics signatures into curated biological pathways through resources such as Kyoto Encyclopedia of Genes and Genomes (KEGG) and Reactome, providing mechanistic interpretability (Kanehisa, 2002; Jassal et al., 2020). More recent advances make use of machine learning, including random forests and deep learning, which can model non-linear relationships and high-dimensional feature interactions characteristic of multi-omics datasets (Libbrecht and Noble, 2015; Abbasi et al., 2024). Together, this integration of omics layers provides a resource for dissecting complex traits and guiding precision breeding and management strategies (Yang et al., 2017).

**FIGURE 2:**
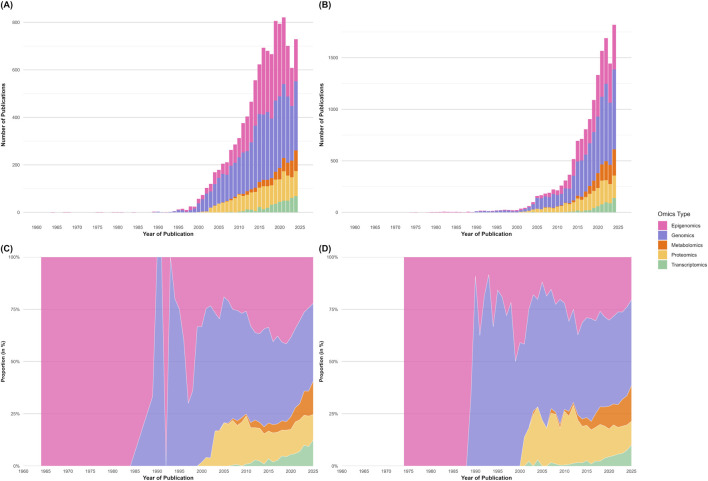
Trends and relative proportions of publications applying omics technologies in mini-livestock research. PubMed indexed publications were classified into five major omics categories: epigenomics, genomics, transcriptomics, proteomics and metabolomics. The absolute number of publications per year in invertebrates **(A)**, and in vertebrates **(B)** highlights the expansion of mini-livestock omics research, particularly driven by genomic studies. **(C,D)** Depict the relative proportion of each omics discipline over time for invertebrates and vertebrates, illustrating the diversification of research approaches and the increasing prominence of transcriptomic and proteomic methods. Data source: PubMed (accessed 4 December 2025; search queries utilized specific taxonomic terms and common names for all organisms referenced in this review (e.g., “*Hermetia illucens*”, “Black Soldier Fly”), combined with Boolean operators across omics categories).

The technological advances and integrative analytical capabilities have also contributed to the growing adoption of omics-based research in mini-livestock species, with publication numbers increasing steadily since 2010 (Figures 2A,B). In invertebrates, epigenomics was the first omics discipline to be reported, with studies dating back to 1964, whereas transcriptomics was introduced much later, with the first publications appearing in 2007. In vertebrates, the first record in the field was in epigenomics in 1974, and transcriptomics was introduced as the latest field in 2002. Beyond these initial milestones, the composition of the field has shifted considerably. The relative contribution of functional omics, most notably transcriptomics and proteomics, has increased markedly in recent years, as illustrated in Figures 2C,D. However, studies focused on mini-livestock still represent only a small fraction of the approximately 2.5 million omics-related publications currently indexed in PubMed (https://pubmed.ncbi.nlm.nih.gov/), with genomic studies making up the majority of this research. With this growing body of research on omics technologies and their potential applications, we can now examine how these approaches are being applied to different mini-livestock groups. Among them, insects have emerged as a particularly well-studied category, with numerous omics investigations providing deep insights into their physiology, genetics, and potential for sustainable production.

## 4 Omics in invertebrates

Invertebrates, particularly those considered as mini-livestock, have become a major focus of recent omics research due to their potential for sustainable protein production. Among them, edible insects have been studied extensively, revealing key adaptations and breeding opportunities (Table 1). In this review, species are presented according to the extent of available scientific literature and ongoing research activities, which is influenced by industrial relevance, and does not reflect any intended prioritization by the authors. For example, the black soldier fly (BSF, *Hermetia illucens*), has emerged as a model species due to its nutritional profile, rearing efficiency, and strong potential as a sustainable protein source (Rumpold and Schlüter, 2013; Huang et al., 2019; Zhan et al., 2020). Subsequently, a novel chromosome-level genome assembly was generated in BSF using long reads, linked short reads and chromatin conformation data (Hi-C) (Generalovic et al., 2021). In addition, another research group constructed a high-quality genome from BSF based on short-read sequencing data and highlighted its findings of 50 antimicrobial peptides, the largest antimicrobial peptide family identified in insects to date, as well as a set of core microbiota suggesting a targeted adaptation of BSF to a pathogen-rich environment and digestion of organic waste (Zhan et al., 2020). Using these reference genomes, multiple studies performed WGS to monitor inbreeding of populations and applied genomic information for commercial breeding programs (Cai et al., 2024). To construct mitochondrial assemblies, whole genome shotgun sequencing was performed, enabling the reconstruction of phylogenetic relationships among major lineages and revealing their long-term evolution with low genomic diversity (Guilliet et al., 2022). Furthermore, WGS-based annotations of gene and protein functions of different BSF strains highlighted major metabolic gene functions and pathways involved in nutrient and energy metabolism (Sukmak et al., 2024). Similarly, initial insights into the genomic potential of the house cricket (*Acheta domesticus)* as a food source and for broader applications were provided by sequencing and assembling a reference genome using both long-read sequencing, Chicago libraries, and Hi-C data (Dossey et al., 2023). Furthermore, sequencing, assembly and annotation of the Mediterranean field cricket (*Gryllus bimaculatus*) genome, and its comparison to the Hawaiian cricket (*Laupala kohalensis)* and other insects, revealed that hemimetabolous (incomplete metamorphosis) genomes have expanded largely through transposable element activity (Ylla et al., 2021). In the yellow mealworm (*Tenebrio molitor*), long and short reads and long-range data obtained from a male pupa, transcripts from 12 different life stages/sexes, as well as an adult individual’s head, highlighted the challenge posed by a comparatively large genome with mostly homogeneous satellite DNA sequences of high copy numbers, and provided a framework for future genomics studies (Eriksson et al., 2020; Eleftheriou et al., 2021; Oppert et al., 2023). A similar approach was used to build a reference genome for the superworm (*Zophobas morio* = *Zophobas atratus*) and a further independent reference for the yellow mealworm (Kaur et al., 2023). Subsequent comparison of both genomes revealed extensive macrosynteny across the family *Tenebrionidae*, as well as numerous within-chromosome rearrangements (Kaur et al., 2023). An even higher level of reference genome completeness was reached in the silkworm (*Bombyx mori)*, for which researchers produced a telomere-to-telomere assembly using long-read sequencing technologies as well as a reference transcriptome (Kawamoto et al., 2019; Yokoi et al., 2021; Zhang T. et al., 2022). In the earthworm (*Eisenia andrei*), researchers produced a chromosome-level reference genome, a large-scale transcriptome and single-cell RNA-sequencing data to investigate the cause for its strong regenerative ability (Shao et al., 2020). Furthermore, *de novo* assembly methods were applied in the honey bee (*Apis mellifera/dorsata/cerana*) to produce draft genomes, which were used to support research in the field of social communication, defensive aggression and scouting behaviour (Elsik et al., 2014; Park et al., 2015; Southey et al., 2016; Oppenheim et al., 2020). Functional genomic analysis revealed 60 genomic variants associated with scout and recruit behavioural castes, within 39 genes corresponding with neuronal function, exoskeleton, immune response, salivary gland development and enzymatic food processing (Southey et al., 2016). In addition, genomic sequences from modern and historic honey bee populations were studied for potential genetic bottlenecks, selection signatures and diversity (Wragg et al., 2016; Parejo et al., 2020; Minozzi et al., 2021; Chen C. et al., 2022).

**TABLE 1:** Overview of mini-livestock studies in the field of omics focusing on insects. The table provides a comprehensive summary of omics studies identified for insects, detailing the omics category, sequencing methodology, targeted biological features, and overall study design.

Investigated mini-livestock species	Omics field (“-omics”)	Sequencing-technique	Targets and study design	References
Black Soldier Fly *(Hermetia illucens)*	Gen-, Epigen-	WGS, Hi-C	High-quality chromosome-level genome assembly, inbreeding events, functional characterization of genes	Generalovic et al. (2021)
Black Soldier Fly *(Hermetia illucens)*	Gen-	WGS	Phylogenetic analysis, cryptic genetic and genomic diversity over five continents, adaptation to different lineages, mitochondrial genome	Guilliet et al. (2022)
Black Soldier Fly *(Hermetia illucens)*	Gen-	WGS	Differentiation of two captive populations, implementation of a breeding program, marker set, monitoring of inbreeding	Cai et al. (2024)
Black Soldier Fly *(Hermetia illucens)*	Gen-	WGS	Nutrients and energy, functions of protein-coding genes, metabolic capability and versatility, insights into transforming waste	Sukmak et al. (2024)
Black Soldier Fly *(Hermetia illucens)*	Gen-, Transcript-	WGS, RNA-seq	High-quality genome, CRISPR/Cas9-based gene editing approach for flightless and enhanced feeding capacity phenotypes, optimizing BSF lines for industrialization and better natural waste recycling, functional gene annotation and pathway-enrichment	Zhan et al. (2020)
Black Soldier Fly *(Hermetia illucens)*	Prote-, Lipid-	nanoLC-MS/MS, GC-MS	Mass spectrometry for proteomic and lipidomic analysis, non-food potential of oil and proteins from insects	Rabani et al. (2019)
Black Soldier Fly *(Hermetia illucens)*	Transcript-, Prote-	RNA-seq, LC-MS/MS	Analysis of cold- and heat-tolerant larvae, differential gene expression analysis, changes in bodyweight, survival rate and metabolism	Feng et al. (2024b)
Black Soldier Fly *(Hermetia illucens)*	Metabol-	LC-MS	Larvae feed utilization rate after UV treatment	Lu et al. (2022a)
Black Soldier Fly *(Hermetia illucens)*	Transcript-	RNA-seq	*De novo* transcriptome sequencing, crude fat accumulation mechanisms, usage for insectile biodiesel	Zhu et al. (2019)
Black Soldier Fly *(Hermetia illucens)*	Transcript-	RNA-seq	Investigation of lysozymes, antimicrobial activity	Zhang et al. (2022c)
Black Soldier Fly *(Hermetia illucens)*	Transcript-, Metabol-	RNA-seq, LC-MS/MS	Effect of pig manure and swill on the transcriptome and metabolome, GO and KEGG enrichment analysis, optimizing substrate selection	Zhang et al. (2024a)
Cricket *(Gryllus veletis and Gryllus lineaticeps)*	Transcript-	RNA-seq	Assembling of the complete mitochondrial genomes, phylogenetic analysis	Torson et al. (2022)
Cricket *(Gryllus spp.& Acheta domesticus)*	Transcript-	RNA-seq	Draft transcriptome, analysis of life stages in different species	Oppert et al. (2020)
Cricket *(Acheta domesticus)*	Transcript-	RNA-seq	Genes associated with flight muscle histolysis	Lu et al. (2022b)
Cricket – Asian *(Teleogryllus occipitalis)*	Gen-	WGS	Draft genome, mitochondrial genome assembly and annotation	Kataoka et al. (2020)
Cricket *(Acheta domesticus)*	Gen-	WGS	High-quality annotated genome assembly, gene groups related to immunity, CRISPR/Cas9-approaches, developing technologies for downstream commercial applications	Dossey et al. (2023)
Cricket *(Gryllus bimaculatus) and (Laupala kohalensis)*	Gen-, Transcript-	WGS, RNA-seq	Comparing two different species, genome assembly and annotation, methylation levels	Ylla et al. (2021)
Earthworm *(Eisenia andrei)*	Gen-, Transcript-	WGS, RNA-seq, scRNA-seq	*De novo* genome assembly, genome annotation, large-scale transcriptome and single-cell sequencing during regeneration	Shao et al. (2020)
Hazelnut weevil *(Curculio dieckmanni)*	Transcript-	RNA-seq	Upregulation of immune-related transcripts, antimicrobial peptides, stress-responsive proteins (e.g., Heat shock protein HSP70), metabolic reprogramming marked by the downregulation of carbohydrate metabolic pathways, energy conservation mechanisms	Wang et al. (2025)
Honey bee – Asian *(Apis cerana)*	Gen-	WGS	*De novo* assembly methods, draft genome, social insect communication, behaviour, evolutionary trajectory	Park et al. (2015)
Honey bee – Asian *(Apis dorsata and Apis mellifera)*	Gen-	WGS	*De novo* assembly, comparison of the two subspecies, and candidate genes for defensive aggression traits	Oppenheim et al. (2020)
Honey bee – Drones *(Apis mellifera)*	Gen-	WGS	Sequencing drones, genomic selection for royal jelly	Wragg et al. (2016)
Honey bee – Italian *(Apis mellifera ligustica)*	Gen-	WGS	Population structure on the Italian peninsula, pattern of genetic variation, morphometric analysis, limited genetic introgression from other breeds	Minozzi et al. (2021)
Honey bee – Swiss/Western *(Apis mellifera mellifera)*	Gen-	WGS	Sequencing individuals from natural history museum, genetic bottlenecks and diversity, selection signatures between modern and historic bees	Parejo et al. (2020)
Honey bee – Western *(Apis mellifera)*	Transcript-	RNA-seq	Meta-Analysis of public RNA-Seq data, construction of reference transcriptome	Yokoi et al. (2022)
Honey bee – Western *(Apis mellifera mellifera)*	Gen-	WGS	Variant detection and genetic variability, association between genomic variants and scouting behaviour, genes for neuronal function, exoskeleton, immune response, salivary gland development and enzymatic food processing	Southey et al. (2016)
Honey bee *(Apis mellifera ligustica)*	Gen-	WGS	Genetic diversity, population structure	Chen et al. (2022a)
Honey bee *(Apis mellifera lineaus)*	Transcript-	RNA-seq	Sublethal effects of insecticides (neonicotinoid thiamethoxam) on the transcriptome, pathway analysis	Shi et al. (2017)
Honey bee *(Apis mellifera)*	Gen-	WGS	Re-sequencing of highland and lowland bees, candidates for adaptation to highland habitats	Wallberg et al. (2017)
Honey bee *(Apis mellifera)*	Gen-, Transcript-	WGS, RNA-seq	Improved assembly, *de novo* annotation, improved gene prediction	Elsik et al. (2014)
Honey bee *(Apis mellifera)*	Transcript-	miRNA-seq	Social behaviour in nurse bees, miRNAs in the brain, differential miRNA expression	Greenberg et al. (2012)
Honey bee *(Apis mellifera)*	Transcript-	RNA-seq	Comparison between queen and worker destined larvae, differential gene expression analysis	Chen et al. (2012)
Honey bee *(Apis mellifera)*	Transcript-	RNA-seq	Brains of honey bee queens	Manfredini et al. (2015)
Honey bee *(Apis mellifera)*	Transcript-	RNA-seq	Molecular effects of cypermethrin, functional analysis of muscular development, structure and function, physiological consequences	Fent et al. (2020)
Honey bee *(Apis mellifera)*	Transcript-	RNA-seq	Sex determination of queen, worker and drone larvae, different pattern of gene expression regulation during the larval development	He et al. (2019b)
Honey bee *(Apis mellifera)*	Transcript-	RNA-seq	Nosema ceranae (parasite) infection, reduced immune function	Badaoui et al. (2017)
Honey bee *(Apis mellifera)*	Transcript-	RNA-seq	Investigation of p-coumaric acid, gene regulation in caste determination, diet of queen-destined larvae	Mao et al. (2015)
Honey bee *(Apis mellifera)*	Transcript-, Epigen-	RNA-seq, BS-seq	Bisulfite sequencing (BS-seq), Israeli Acute Paralysis Virus (IAPV) infection, fat bodies, two distinct molecular pathways, mediated by transcription and methylation	Galbraith et al. (2015)
Honey bee *(Apis mellifera)*	Transcript-	scRNA-seq	Mapping of cell types to different developmental stages of the worker honey bee	Patir et al. (2023)
Honey bee *(Apis mellifera)*	Transcript-	scRNA-seq	Distinct gene expression in the brains of queens and workers	Zhang et al. (2022e)
Honey bee *(Apis mellifera)*	Transcript-	RNA-seq, scRNA-seq	Single-cell transcriptomics and gene regulatory network analyses, bee aggression	Traniello et al. (2023)
Honey bee *(Apis mellifera)*	Transcript-	snRNA-seq, spatial transcriptomics	Expression patterns of brain cells associated with the behavioural maturation from nursing to foraging	Mu et al. (2025)
Honey bee *(Apis mellifera)*	Transcript-, Epigen-	RNA-seq, WGBS	Whole-genome bisulfite sequencing (WGBS), parent-of-origin effects manifested in both DNA methylation and gene expression	Wu et al. (2020b)
Palm Weevil (Rhynchophorus ferrugineus)	Transcript-	RNA-seq	*De novo* transcriptome, differential gene expression, peroxisome pathway associated with metabolic pathways, material transportation and organ tissue formation	Yang et al. (2020a)
Silkworm *(Bombyx mori)*	Gen-, Epigen-	WGS, Hi-C	*De novo* genome assembly, telomere-to-telomere reference genome, synteny	Zhang et al. (2022d)
Silkworm *(Bombyx mori)*	Epigen-	MethylC-Seq	Silkworm domestication, differential analysis of methylomes	Xiang et al. (2013)
Silkworm *(Bombyx mori)*	Gen-, Transcript-	WGS, RNA-seq	High-quality *de novo* genome assembly, prediction and identification of gene families and repetitive elements, gene prediction	Kawamoto et al. (2019)
Silkworm *(Bombyx mori)*	Gen-, Epigen-, Transcript-	WGS, RNA-seq, WGBS	Genome, transcriptome and WGBS sequencing analysis, multi-omics approach, silkworm heterosis	Xu et al. (2022a)
Silkworm *(Bombyx mori)*	Transcript-	RNA-seq	Transcriptome study at different developmental stages	Li et al. (2012)
Silkworm *(Bombyx mori)*	Transcript-	RNA-seq	Susceptibility and resistance to fungal infection of different strains	Xing et al. (2017)
Silkworm *(Bombyx mori)*	Transcript-	RNA-seq	Susceptibility and resistance to fungal infection of different strains	Luan et al. (2018)
Silkworm *(Bombyx mori)*	Transcript-	RNA-seq	Differential gene expression associated with lipopolysaccharide-induced immune priming	Yi and Wu (2024)
Silkworm *(Bombyx mori)*	Transcript-	RNA-seq	Expression changes of the fat body in response to selenium treatment, differentially expressed genes in lipid metabolism and antioxidant defense	Jiang et al. (2020)
Silkworm *(Bombyx mori)*	Transcript-	RNA-seq	Hydrogen sulfide exposure, genes involved in endocytosis, glycolysis/gluconeogenesis, citrate cycle, synthesis of fibroin	Zhang et al. (2021a)
Silkworm *(Bombyx mori)*	Transcript-	RNA-seq	Differentially expressed genes under different temperature conditions	Guo et al. (2018)
Silkworm *(Bombyx mori)*	Transcript-	RNA-seq	Differentially expressed genes in wild versus domestic individuals, immune response, antioxidant systems	Fang et al. (2015)
Silkworm *(Bombyx mori)*	Transcript-	RNA-seq	Differentially expressed genes associated with cocoon and silk yields	Li et al. (2016)
Silkworm *(Bombyx mori)*	Transcript-	RNA-seq	Lead-induced detoxification-related genes	Ye et al. (2024)
Silkworm *(Bombyx mori)*	Transcript-	RNA-seq	Transcriptomics of the anterior silk gland, ion transportation, energy metabolism, protease inhibitors and cuticle proteins involved in the process of silk formation and spinning	Chang et al. (2015)
Silkworm *(Bombyx mori)*	Transcript-	RNA-seq	Differential expression of genes between diapause-inducing and non-diapause-inducing groups	Chen et al. (2017)
Silkworm *(Bombyx mori)*	Transcript-	RNA-seq	Long-non-coding RNAs, hub lncRNAs as regulators of biosynthesis, translocation, and secretion of silk proteins	Wu et al. (2016)
Silkworm *(Bombyx mori)*	Transcript-	RNA-seq	Dosage compensation	Gopinath et al. (2017)
Silkworm *(Bombyx mori)*	Transcript-	RNA-seq	Dimethoate exposure on eggs, silkworm reproduction	Zheng et al. (2022)
Silkworm *(Bombyx mori)*	Transcript-	RNA-seq	Immune priming in haemocytes	Yi et al. (2019)
Silkworm *(Bombyx mori)*	Transcript-	RNA-seq	Immune response to bidensovirus infection, antioxidant genes	Sun et al. (2020)
Silkworm *(Bombyx mori)*	Transcript-	RNA-seq	High temperature and humidity	Xiao et al. (2017)
Silkworm *(Bombyx mori)*	Transcript-	RNA-seq	Thermal parthenogenesis, differentially expressed genes related to reactive oxygen species removal, DNA repair and heat shock response	Liu et al. (2015)
Silkworm *(Bombyx mori)*	Transcript-	RNA-seq	Ovaries, differentially expressed genes involved in metabolism, genetic information processing, environmental information processing, cellular processes and organismal systems	Xue et al. (2015)
Silkworm *(Bombyx mori)*	Transcript-	RNA-seq	Time-course transcriptome expression data of parts of the silk gland	Masuoka et al. (2024)
Silkworm *(Bombyx mori)*	Transcript-	RNA-seq	Reference transcriptome, expression analysis and profiling, transcriptional factor genes	Yokoi et al. (2021)
Silkworm *(Bombyx mori)*	Transcript-	RNA-seq	Naked pupa mutant, cellular stress responses	Hu et al. (2019)
Silkworm *(Bombyx mori)*	Transcript-	RNA-seq	Treatment with titanium dioxide nanoparticle, effect on silk gland, enrichment of metabolic pathway-related genes	Xue et al. (2017a)
Silkworm *(Bombyx mori)*	Transcript-	RNA-seq (long reads)	Transcriptome atlas of silk glands using single-molecule long-read sequencing	Chen et al. (2020)
Silkworm *(Bombyx mori)*	Transcript-	RNA-seq (long reads)	Improved gene annotation, differentially expressed genes for midgut related to digestive enzyme production, transmembrane transport, chitin metabolism, and hormone regulation	Shen et al. (2022)
Silkworm *(Bombyx mori)*	Transcript-	RNA-seq, scRNA-seq	Classification of silkworm hemocytes under baculovirus-infection, infection suppresses the RNA interference and immune response	Feng et al. (2021a)
Silkworm *(Bombyx mori)*	Transcript-	RNA-seq, scRNA-seq	Single-cell transcriptomic atlas of the silk-producing organ	Ma et al. (2022)
Silkworm *(Bombyx mori)*	Transcript-	RNA-seq, snRNA-seq, spatial transcriptomics	Single-nucleus and spatial transcriptomics atlas of silk-secreting organs	Ma et al. (2024)
Silkworm *(Bombyx mori)*	Transcript-	scRNA-seq	Hemocyte clusters, broad division of hemocytes in granulocytes, plasmatocytes, oenocytoids	Feng et al. (2022)
Silkworm *(Bombyx mori)*	Transcript-	snRNA-seq	Single-nucleus sequencing of brain hemocytes, cell subsets with antiviral function	Feng et al. (2024a)
Silkworm *(Bombyx mori)*	Transcript-	snRNA-seq	Single-nucleus sequencing of fat body, response to nucleopolyhedrovirus infection	Feng et al. (2023)
Silkworm *(Bombyx mori)*	Transcript-	scRNA-seq	Brain cell repertoire, individual neuropeptide expression	Liu et al. (2024b)
Silkworm *(Bombyx mori)*	Prote-	Tandem-MS	Comparative analysis, diverse functions and dynamic changes, silk protein compositions	Dong and Chen (2013)
Silkworm *(Bombyx mori and Philosamia cynthia ricini)*	Prote-	MADLI-TOF MS	Silk gland proteins, phosphate residues	Zhang et al. (2006)
Silkworm *(Bombyx mori)*	Prote-	nanoLC-MS/MS	Comparative analysis of silk gland, ribosome biogenesis, protein identification and quantification, protein synthesis	Li et al. (2015)
Superworm *(Zophobas atratus)*	Gen-	WGS	Complete mitochondrial genome, phylogenetic analysis	Bai et al. (2019)
Superworm *(Zophobas atratus)*	Transcript-	RNA-seq	*De novo* transcriptome and functional genome annotation, prediction of antimicrobial peptides and haemolytic activity	Lee et al. (2021)
Superworm *(Zophobas atratus)* Yellow mealworm *(Tenebrio molitor)*	Gen-	WGS	High-quality genome assemblies, gene prediction, phylogenomic and synteny analysis	Kaur et al. (2023)
Yellow Mealworm *(Tenebrio molitor)*	Gen-, Transcript-	WGS, RNA-Seq	Genome assembly based on combination of long, short reads and Hi-C data	Eleftheriou et al. (2021)
Yellow Mealworm *(Tenebrio molitor)*	Gen-	WGS	*De novo* genome assembly, mitochondrial genome	Eriksson et al. (2020)
Yellow Mealworm *(Tenebrio molitor)*	Gen-, Epigen-, Transcript-	WGS, Hi-C, RNA-seq	Assembly, gene prediction, gene annotation, CRISPR-Cas gene editing, life stage-specific transcriptomes	Oppert et al. (2023)
Yellow Mealworm *(Tenebrio molitor)*	Transcript-	RNA-seq	Gene expression profiling, identify chitin coding genes, coding enzymes, polygenetic dividing, biological functions	Li et al. (2022b)
Yellow Mealworm *(Tenebrio molitor)*	Transcript-	RNA-seq	Sequencing with focus on immune response genes to parasitisation, molecular host-parasitoid interaction	Zhu et al. (2013)
Yellow Mealworm *(Tenebrio molitor)*	Transcript-	RNA-seq	Immune gene expression after infection, identifying defence mechanisms, pathway analysis	Johnston et al. (2013)
Yellow Mealworm *(Tenebrio molitor)*	Transcript-, Prote-	RNA-seq, MALDI-TOF MS	Comparison of neuropeptides and transcriptomic data, *de novo* sequencing, gene bank (neuropeptidomics)	Marciniak et al. (2022)

As a result, genomic studies have provided a comprehensive understanding of the genetic code, whereas epigenetic research has elucidated the complex interplay between environmental factors and gene expression in insect mini-livestock. The utilization of insects as model organisms in epigenetic studies has yielded significant insights into the epigenetic mechanisms underlying disease susceptibility and transgenerational inheritance patterns (Mukherjee et al., 2015). For example, studies on silkworm revealed the role of DNA methylation in rapid phenotypic adaptation mediated by DNA methyltransferase 1 (DNMT1), which might have contributed to the domestication of this species (Xiang et al., 2013; Wang X. et al., 2021). However, on the level of gene expression, transcriptomics studies showed that modern breeding potentially had a stronger selection effect on silk yield traits and pathogen tolerance in the silkworm than its domestication (Fang et al., 2015; Li et al., 2016; Luan et al., 2018). Furthermore, RNA-seq data from larvae provided an insight into the resistance and susceptibility of different strains against fungal, virus or bacterial infection, thermo-tolerance, diapause preparation, detoxification mechanisms and antioxidant defence, highlighting the complexity of the silkworm transcriptome (Li et al., 2012; Chen et al., 2017; Xiao et al., 2017; Xing et al., 2017; Guo et al., 2018; Jiang et al., 2020; Sun et al., 2020; Zhang R. et al., 2021; Ye et al., 2024; Yi and Wu, 2024). In contrast to protein-coding mRNAs, long non-coding RNAs were postulated to be even more specific for different silkworm tissues and were found to be involved as regulators of the biosynthesis, translocation, and secretion of silk proteins (Wu et al., 2016). At even higher resolution, single cell sequencing of silkworm haemocytes revealed a high level of specialization of these cells and showed a significant effect of RNA interference (RNAi) suppression induced by a baculovirus infection (Feng M. et al., 2021; Feng et al., 2022). A similar strategy was used to build long-read-based transcriptome and single-cell transcriptome atlases of the silk gland, which offered a comprehensive and detailed understanding of its function and regulation (Chen et al., 2020; Ma et al., 2022). This was even enhanced by a spatiotemporal transcriptomic atlas of the silk glands, and multi-omics approaches, providing a valuable reference for elucidating the mechanism of efficient silk protein synthesis (Xu H. et al., 2022; Ma et al., 2024). On the protein level, silk gland development and silk protein protection and compositions further completed the picture (Zhang et al., 2006; Dong et al., 2013; Li et al., 2015).

Similar to the silkworm, functional omics studies in BSF were aimed at the non-food potential of this species. *De novo* transcriptome sequencing has identified genes involved in fat metabolism, thereby contributing to more economical BSF-based biodiesel production (Zhu et al., 2019). In contrast, transcriptome, metabolome and proteome analyses of BSF larvae have elucidated bioconversion performance under different temperature conditions and dependent on the type of organic waste, as well as highlighted an association of UV light treatment of larvae with the generation of functional proteins and bioactive compounds (Lu J. et al., 2022; Zhang S. et al., 2022; Feng X. et al., 2024). It was found that the active intestinal microbes and their functional genes in the BSF gut microbiome delineated the genetic variability in wild-collected and domesticated BSF populations from different continents and showed a response to high concentrations of antibiotics (Khamis et al., 2020; Pei et al., 2023).

In the yellow mealworm, gene expression profiling was particularly carried out to learn more about tissue- or developmental stage-specific genes and their potential function, identifying enzymes involved in chitin metabolism, parasitoid-induced immune-related genes, and factors underlying long-lasting immune response to bacterial challenge (Johnston et al., 2013; Zhu et al., 2013; Li L. et al., 2022). Similarly, a *de novo* transcriptome assembly and functional annotation in the superworm was used to predict antimicrobial peptides and haemolytic activity (Lee et al., 2021). Both yellow mealworm and superworm also underwent transcriptomic and mass spectrometry analyses of the central nervous system to identify neuropeptides and neuropeptide-like and protein hormones (Marciniak et al., 2022). In general, the brain has been a target of various insect species studies, due to the interest in socially regulated behaviour such as division of labour among honey bees. For example, mRNA or microRNA expression levels in the honey bee brain have been analyzed for their role in behavioural specialization of adult workers or queen (Greenberg et al., 2012; Manfredini et al., 2015). Single-nucleus RNA sequencing and spatial transcriptomics of the honey bees brain revealed expression patterns of brain cells associated with the behavioural maturation from nursing to foraging (Mu et al., 2025). In addition, honey bee caste differentiation was investigated using high-throughput RNA-Seq of larvae (Chen et al., 2012; Mao et al., 2015; He X. J. et al., 2019). More recently, single cell RNA sequencing (scRNA-seq) has been applied to identify caste differentiation-related factors in the queen and to map cell types across developmental stages (prepupa at day 11 and pupa at day 15) of worker honey bees (Zhang W. et al., 2022; Patir et al., 2023). Furthermore, the impact of numerous biotic stressors on honey bees has been extensively studied using functional omics methods; Researchers employed RNA-seq to characterize immune responses to parasite infection or insecticide exposure, and examined methylation patterns in the fat body linked to virus infection (Galbraith et al., 2015; Badaoui et al., 2017; Shi et al., 2017; Fent et al., 2020). Similarly, immune responses were studied in the red palm weevil (*Rhynchophorus ferrugineus*), with a primary focus on effective pest-management through potential gene knockdowns, although its possible application as a food source was also considered (Yang H. et al., 2020; Fernando et al., 2023).

Although most of these above-mentioned studies focused on individual research questions and species, efforts have also been made to analyse omics data across species; For example, High-Performance Liquid Chromatography–Tandem Mass Spectrometry (HPLC-MS/MS) technology was applied to construct a novel integrated metabolic database for nine insect species across three metamorphosis types identifying 1,442 metabolites (Li et al., 2023). The study by Li *et al.* revealed significantly enriched pathways, including ABC transporters and tyrosine metabolism, thereby creating a valuable reference that enhances our understanding of insect metabolic evolution and adaptation (Li et al., 2023). Genome assemblies of insects and further omics data have been collected in the InsectBase (http://www.insect-genome.com), which has made significant progress by storing more than 16 million sequences from 815 species to date (Yin et al., 2016; Mei et al., 2022). However, this represents only a small fraction of the 2,205 insects classified as edible (Omuse et al., 2024), highlighting the need for greater efforts to gather more comprehensive data on these species. Modern urban insect farming projects, such as small-scale cricket rearing initiatives in Kenya, BSF bioconversion facilities in Singapore, and a yellow mealworm pilot production in the Netherlands, demonstrated how insect breeding could be integrated into future city environments to supply sustainable protein and manage organic waste streams (Ayieko et al., 2016; Dalton and Al-Zubiedi, 2019; Ramzy et al., 2025).

Beyond insects, other invertebrate groups such as molluscs (Table 2) are also gaining attention, particularly because of their central role in aquaculture and their unique physiological adaptations revealed by omics studies (Klein et al., 2019). As with insects, several mollusc species were already characterized through *de novo* genome sequencing and chromosome-level assemblies (Liu C. et al., 2018; Guo et al., 2019; Chueca et al., 2021; Ma et al., 2023; Ishii et al., 2025). The combination of short reads and long reads with Hi-C sequences and a transcriptome for annotation substantially improved assembly quality, surpassing that of most other *Panpulmonata* proteomes (Guo et al., 2019; Ma et al., 2023; De Jode et al., 2024; Ishii et al., 2025). Thus, in the rough periwinkle (*Littorina saxatilis/arcana*), WGS and mapping to the annotated genome allowed to study inversion polymorphisms often widespread across the species and associated with rapid parallel adaptation to heterogeneous environments (Morales et al., 2019; Reeve et al., 2024). Similarly, the rapid adaptive capacity of the invasive apple snail (*Pomacea canaliculate*) to diverse environments was allocated to its high genetic diversity studied by different omics techniques (Lu et al., 2024). Furthermore, sequencing of the transcriptome and small RNA sequencing (sRNA-seq) in the Asian tramp snail (*Bradybaena similaris)* highlighted genes and regulatory elements involved in xenobiotic metabolism (Yang Q. et al., 2020). This complex system for metabolizing xenobiotics was also found to be affected by arsenic pollution in the apple snail, displaying a dose-dependent effect on growth (Bi et al., 2024). To learn more about the genetics of the family of apple snails, the *Ampullariidae*, a transcriptome database was generated for seven subspecies using WGS and previous RNA-seq data (Ip et al., 2018). Subsequently, taking full advantage of this database, a multi-omics approach was applied to study the perivitelline fluid proteome from apple snail eggs, highlighting the adaptive capacities of different subspecies (Ip et al., 2019).

**TABLE 2:** Overview of mini-livestock studies in the field of omics focusing on molluscs. The table provides a comprehensive summary of omics studies identified for molluscs, detailing the omics category, sequencing methodology, targeted biological features, and overall study design.

Investigated mini-livestock species	Omics field (”-omics”)	Sequencing-technique	Targets and study design	References
Apple Snail *(Pomacea canaliculata)*	Gen-	WGS	*De novo* genome assembly at chromosome level, transposable elements, cellular homeostasis system, invasiveness	Liu et al. (2018a)
Apple Snail *(Pomacea canaliculata)*	Gen-	WGS, RNA-seq, Hi-C	*De novo* genome assembly, gene annotation, genes associated with low temperature adaption	Lu et al. (2024)
Apple Snail *(Pomacea canaliculata)*	Transcript-, Metabol-	RNA-seq, LC-MS/MS	Arsenic pollution, stress response in freshwater snails	Bi et al. (2024)
Apple Snail *(Pomacea canaliculata)*	Transcript-	RNA-seq	*De novo* transcriptome assembly of different genera of *Ampullariidae*, development of AmpuBase database	Ip et al. (2018)
Apple Snail *(Pomacea canaliculata)*	Prote-	LC-MS/MS	Perivitelline fluid proteomes, aquatic and aerial egg-laying *Ampullariidae*, selection events, evolutionary transition	Ip et al. (2019)
Asian Tramp Snail *(Bradybaena similaris)*	Gen-, Transcript-, Epigen-	WGS, RNA-seq, Hi-C	*De novo* chromosome-level assembly, gene annotation, structural arrangement via Hi-C	Ishii et al. (2025)
Asian Tramp Snail *(Bradybaena similaris)*	Gen-, Transcript-	RNA-seq, sRNA-seq	Xenobiotic metabolism-related genes and potential regulatory miRNA	Yang et al. (2020b)
Chinese Mystery Snail *(Cipangopaludina cathayensis)*	Gen-, Transcript-, Epigen-	WGS, Hi-C, RNA-seq	First chromosome-level genome, anchoring pseudochromosomes with Hi-C, putative genes for evolutionary studies	Ma et al. (2023)
Giant African Land Snail *(Achatina fulica)*	Gen-, Transcript-, Epigen-	WGS, Hi-C, RNA-seq	First *de novo* chromosomal-level genome assembly, k-mer-based method, phylogenetic analysis	Guo et al. (2019)
Roman Snail *(Helix pomatia)*	Transcript-	RNA-seq	Complete mitogenome, phylogeny	Orabek et al. (2019)
Roman Snail (*Helix pomatia*)	Transcript-	RNA-seq	Reference transcriptome, adenosine deaminase-related growth factor	Tzertzinis et al. (2023)
Rough Periwinkle *(Littorina saxatilis)*	Gen-	WGS	Genomics of rapid parallel adaptation in response to two independent environmental axes, chromosomal inversions	Morales et al. (2019)
Rough Periwinkle *(Littorina saxatilis and arcana)*	Gen-	WGS	Whole genome data from 107 snails, chromosomal inversion polymorphisms	Reeve et al. (2024)
Rough Periwinkle *(Littorina saxatilis)*	Gen-, Epigen-, Transcript-	WGS, RNA-seq, Hi-C	Chromosome-scale reference genome	De Jode et al. (2024)
Thyme Snail *(Candidula unifasciata)*	Gen-, Transcript-	WGS, RNA-seq	*De novo* genome assembly, gene annotation	Chueca et al. (2021)

## 5 Omics in vertebrates

Omics technologies have significantly advanced research not only in invertebrates but also in vertebrate species classified as mini-livestock, though with distinct applications and challenges. In particular, mammalian research (Table 3) has benefited substantially from progress in the genomic field (Georges et al., 2019). As noted above for the invertebrates, the emphasis on certain species reflects the current availability of scientific literature and research activity, rather than a prioritization by the authors.

**TABLE 3:** Overview of mini-livestock studies in the field of omics focusing on mammals. The table provides a comprehensive summary of omics studies identified for mammals, detailing the omics category, sequencing methodology, targeted biological features, and overall study design.

Investigated mini-livestock species	Omics field (“-omics”)	Sequencing-technique	Targets and study design	References
Cane Rat *(Thryonomys swinderianus)* Guinea Pig *(Cavia porcellus)*	Metabol-, Transcript-	UHPLC-MS/MS, RNA-Seq	Ultra-High-Performance Liquid Chromatography –Tandem Mass Spectrometry (UHPLC-MS/MS), common molecular markers and pathways between rats and guinea pigs in response to liver toxicity	Schyman et al. (2021)
Capybara *(Hydrochoerus hydrochaeris)*	Gen-	WGS	Gigantism, phylogenetic pathways, adaptive protein evolution, postnatal bone growth	Herrera-Alvarez et al. (2021)
Capybara *(Hydrochoerus hydrochaeri)*	Gen-	WGS	Draft genome	Babarinde and Saitou (2020)
Capybara *(Hydrochoerus hydrochaeris)* Guinea Pig *(Cavia porcellus) (Cavia aperea)*	Gen-	WGS (from archive)	Mitochondrial analysis, phylogenetic inference, pattern identification, comparison with guinea pigs	da Silva et al. (2024)
Guinea Pig *(Cavia aperea)*	Gen-, Epigen-	WGS, MEBS	Methylated DNA Enrichment by Binding and Sequencing (MEBS), methylome analysis, DNA enrichment	Weyrich et al. (2014)
Guinea Pig *(Cavia porcellus)*	Gen-	WGS (from archive)	Phylogenetics, immunoglobulin heavy and light chain genes, antibody diversity	Guo et al. (2012)
Guinea Pig *(Cavia porcellus)* Rabbit *(Oryctolagus cuniculus)*	Transcript-	RNA-seq	Brain gene expression level of domesticated versus wild animals	Albert et al. (2012)
Miniature pig *(Sus Scrofa domesticus)*	Gen-	WGS	Comparison of the genomic sequence of microminipigs, small-scale SNPs and translational modifications, large-scale deletion and insertion polymorphisms	Miura et al. (2014)
Miniature pig *(Sus Scrofa domesticus)*	Gen-, Transcript-	WGS, RNA-seq	Population genomics in different minipigs (Bama, Göttingen, Mini-LEWE, Wuzhishan, Yucatan, Korean, Minnesota), *PLAG1*, *CHM*, and *ESR1* are candidate key genes for body size determination	Kwon et al. (2024)
Miniature pig *(Sus Scrofa domesticus)*	Metabol-	LC-MS	Liquid Chromatography–Tandem Mass Spectrometry (LC-MS/MS), postprandial metabolic responses, significant amino acids and phospholipids significant in metabolism after food intake	Polakof et al. (2015)
Miniature pig – Bama minipig *(Sus scrofa domesticus)*	Gen-, Epigen-, Transcript-	WGS, RNA-seq, Hi-C	3D genome organization and performed transcriptome characterization of two adipose depots	Zhang et al. (2022b)
Miniature pig – Bama minipig *(Sus scrofa domesticus)*	Gen-, Epigen-, Transcript-	WGS, RNA-seq, Hi-C	Full chromosome-scale reference genome, model for diabetic disease	Zhang et al. (2019)
Miniature pig – Bama minipig *(Sus scrofa domesticus)*	Prote-	LC-MS/MS	Cartilage post-traumatic osteoarthritis of anterior cruciate ligament reconstruction, osteoarthritis-related proteins	Li et al. (2020a)
Miniature pig – Bama minipig *(Sus scrofa domesticus)*	Metabol-	LC-MS/MS	Biomarkers for early diagnosis of atherosclerosis under high-cholesterol and high-fat diet	Jia et al. (2024b)
Miniature pig – Bama minipig *(Sus scrofa domesticus)*	Transcript-	scRNA-seq	Single-cell transcriptomic atlas, unique markers for various tissues and organs	Chen et al. (2025)
Miniature pig – Bama minipig *(Sus scrofa domesticus)*	Transcript-	snRNA-seq	Single-nucleus RNA sequencing (snRNA-seq), transcriptome profiling of Peyer’s patches, mesenteric lymph node, and spleen of germ-free and specific pathogen-free piglet	Miao et al. (2024b)
Miniature pig – Bama minipig *(Sus scrofa domesticus)*	Transcript-	snRNA-seq	Single-cell sequencing of Bama pig testes from fetal stage through infancy, puberty to adulthood	Wang et al. (2024a)
Miniature pig – Bama minipig *(Sus scrofa domesticus)*	Transcript-	scRNA-seq	Single-nucleus RNA sequencing on myocardial samples from both wild-type and metabolic disease-susceptible transgenic pigs	Zhang et al. (2025a)
Miniature pig – Bama minipig, Tibetan pig *(Sus scrofa domesticus)*	Transcript-	RNA-seq	Transcriptome profiling in pituitary gland	Shan et al. (2014)
Miniature pig – Banna miniature pig *(Sus scrofa domesticus)*	Gen-, Epigen-, Transcript-	WGS, RNA-seq, Hi-C	High-quality chromosome-scale reference genome	Chen et al. (2024)
Miniature pig – Diannan small-ear minipig *(Sus scrofa domesticus)*	Gen-	WGS	Genomic characterization, selection signatures for genomics regions affecting meat quality, body size, adaptability, and appetite	Wu et al. (2020a)
Miniature pig – Diannan small-ear minipig *(Sus scrofa domesticus)*	Gen-	WGS	Re-sequencing, mutation for white-point coat colour	Lu et al. (2016)
Miniature pig – Diannan small-ear minipig *(Sus scrofa domesticus)*	Transcript-	RNA-seq, miRNA-seq	Differential gene expression of muscle tissue from Diannan Small-ear, Tibetan pig, Landrace and Yorkshire, key genes for lipid deposition and muscle growth	Wang et al. (2015)
Miniature pig – different types *(Sus Scrofa domesticus)*	Gen-	WGS	Multi-tool copy number detection, miniature pig breeds across different geographical regions	Berghofer et al. (2022)
Miniature pig – Goettingen minipig *(Sus scrofa domesticus)*	Transcript-	scRNA-seq	Pertussis vaccine, antigen-specific humoral and cellular responses, PBMCs	Vaure et al. (2021)
Miniature pig – Goettingen minipig *(Sus scrofa domesticus)*	Transcript-	RNA-seq	Analysis of human antibody transcripts in humanised animals, immunoglobulin gene rearrangements and expression, testing therapeutic approaches	Flisikowska et al. (2022)
Miniature pig – Goettingen minipig *(Sus scrofa domesticus)*	Epigen-	LHC-BS	Liquid Hybridization Capture Bisulfite Sequencing, diet-induced DNA methylation in liver, obesity-related effects	Feng et al. (2021b)
Miniature pig – Goettingen minipig *(Sus scrofa domesticus)*	Prote-	UHPLC/MS	Obesity-related metabolome and gut microbiota	Curtasu et al. (2020)
Miniature pig – Goettingen minipig *(Sus scrofa domesticus)* Tibetan wild boar *(Sus scrofa)*	Gen-, Transcript-	WGS, RNA-seq	Chromosome-level based genome of Göttingen minipig, transcriptomics for tissues with pharmaceutical relevance	Heckel et al. (2015)
Miniature pig – Goettingen minipig Mini-LEWE *(Sus scrofa domesticus)*	Gen-	WGS	Selective sweeps, body size, androgen receptor AR gene as candidate	Reimer et al. (2018)
Miniature pig – Korean minipig *(Sus scrofa domesticus)*	Gen-, Transcript-	WGS, RNA-seq	High-quality chromosome-level genome annotation and assembly	Wy et al. (2024)
Miniature pig – Korean minipig *(Sus scrofa domesticus)*	Gen-, Transcript-	WGS, RNA-seq	Immune response, gene expression patterns, genes as potential biomarkers	Arora et al. (2022)
Miniature pig – Micro-pig from Medi Kinetics *(Sus scrofa domesticus)*	Gen-	WGS	Genes of interest in Micro-pigs, runs of homozygosity analysis	Son et al. (2020)
Miniature pig – MiniLEWE *(Sus scrofa domesticus)*	Transcript-	RNA-seq	Identification of bone marrow-derived mesenchymal stem cell markers, key driver genes for differentiation, differential gene expression analysis	Khaveh et al. (2024)
Miniature pig – Ossabaw *(Sus scrofa domesticus)*	Gen-, Transcript-	WGS, RNA-seq (from database)	*De novo* genome assembly and annotation, leptin receptor has possible significant effects on obesity	Zhang et al. (2021b)
Miniature pig – Wisconsin Miniature Swine *(Sus scrofa domesticus)*	Gen-, Epigen-	WGS, Hi-C	Draft genome assembly	Veith et al. (2025)
Miniature pig – Wuzhishan pig *(Sus scrofa domesticus)*	Gen-	WGS	*De novo* Chinese pig genome, high level of homozygosity	Fang et al. (2012)
Miniature pig – Wuzhishan pig *(Sus scrofa domesticus)*	Epigen-	MeDIP-seq	DNA methylome of blood leukocytes, Chinese indigenous versus Western pig breeds	Yang et al. (2016)
Miniature pig – Yucatan minipig (*Sus scrofa domesticus*)	Gen-	WGS (from database)	Identification of non-synonymous single nucleotide polymorphisms (nsSNPs) in selective sweep regions	Kwon et al. (2019)
Miniature pig – Yucatan minipig *(Sus Scrofa domesticus*	Metabol-	UHPLC–MS/QTOF	Metabolome of serum, urine and liver, diet- and cloning-induced metabolic changes	Curtasu et al. (2019)
Miniature pig – Yucatan minipig *(Sus scrofa domesticus)*	Prote-	LC-MS/MS	Collagen subtypes and crosslinks in different cartilages	Bielajew et al. (2021)
Mole-Rat – African *(Cryptomys hottentotus)*	Transcript-	RNA-seq	Positive selection analysis for candidate genes associated with lifespan	Sahm et al. (2018)
Mole-Rat – African *(Cryptomys hottentotus)*	Epigen-	ChIP-seq, Hi-C (from archive)	Regulatory evolution in the heart and liver tissue, epigenomic profiles, Chromatin Immunoprecipitation Sequencing (ChIP-seq)	Parey et al. (2023)
Rabbit *(Oryctolagus spp.*)	Gen-, Transcript-, Metabol-	RNA-seq, LC-MS/MS	Microbiological regulation with antibiotic-free diet, microbial gene sequencing, differential gene expression and metabolome analysis	Chen et al. (2022b)
Rabbit *(Oryctolagus cuniculus)*	Gen-	SLAF-seq	Genetic variants associated with growth, carcass and meat quality, GWAS	Yang et al. (2020c)
Rabbit *(Oryctolagus cuniculus)*	Prote-	LC-MS/MS	Ageing model system, rabbit liver, liver proteins across age groups, alterations in metabolism affect protein expression	Amin et al. (2020)
Rabbit *(Oryctolagus spp.*)	Gen-	WGS	Resequencing and comparison of wild and domestic rabbits, loci for tame behaviour	Carneiro et al. (2014b)
Rabbit *(Oryctolagus spp.*)	Metagen-	Shotgun metagenome sequencing	Effects of probiotics on growth performance, immunity, intestinal flora and antioxidant capacity, effect of probiotics on health, genome sequencing of microbes	Hou et al. (2024)
Rabbit *(Oryctolagus spp.*)	Metagen-, Metabol-	WMS, LC-MS	WMS (shotgun metagenomic sequencing), longitudinal dynamics of rabbit gut microbiota and host adaptability, functional gene diversity, comparing lipid metabolism in newborns versus adults	Zhao et al. (2024)
Rabbit *(Oryctolagus spp.*)	Metagen-, Transcript-, Metabol	RNA-seq, UHPLC–MS/MS	Microbial metagenomics, transcriptome, and non-targeted metabolomics sequencing of the cecum microflora, differential gene expression analysis	Wang et al. (2022a)
Rabbit *(Oryctolagus spp.*)	Prote-	LC-MS/MS	Comparative and quantitative analysis of colostrum and mature milk, differentially abundant proteins related to immune response and fatty acid metabolism	Huang et al. (2024)
Rabbit *(Oryctolagus spp.*)	Prote-	nanoLC-MS/MS	Characterizing seminal plasma proteins, influence of genetic origin and seasonality	Casares-Crespo et al. (2018)
Rabbit *(Oryctolagus spp.*)	Transcript-, Prote-	RNA-seq, LC-MS/MS	Obese models with high fat diet, differential gene expression analysis	Li et al. (2021)
Rabbit *(Oryctolagus spp.*)	Metabol-	H-NMR metabolomics	Proton Nuclear Magnetic Resonance (H-NMR) metabolomics, influence of early plant saccharide ingestion on gene expression signatures and gut bacteriome and metabolome	Paes et al. (2024)

In livestock species, such as the domestic pig, reference genome sequences have provided a foundation for genetics and genomics research, enabling the use of genetic variants to study breed-specific traits and signatures of selection (Warr et al., 2020; Berghofer et al., 2022). These findings in porcine genetics were not limited to large high-production pigs but could be readily applied to minipigs, offering new opportunities for basic research, medical applications and breeding programs (Wang et al., 2020; Arora et al., 2022; Jia Y. et al., 2024). Research in miniature pigs has led to the creation of further high-quality chromosome level genome assemblies for various breeds (Bama, Banna, Goettingen, Korean, Ossabaw, Wuzhishan, Wisconsin) that have provided a critical framework for genetic studies (Fang et al., 2012; Heckel et al., 2015; Zhang et al., 2019; Zhang Y. et al., 2021; Chen et al., 2024; Wy et al., 2024; Veith et al., 2025). Re-sequencing and mapping to these genomes have enabled the discovery of selection signatures, facilitating the detection of body-size associated genes such as *PLAG1, CHM,* and *ESR1* and further breed- or minipig-specific traits (Miura et al., 2014; Heckel et al., 2015; Lu et al., 2016; Reimer et al., 2018; Kwon et al., 2019; Son et al., 2020; Wu F. et al., 2020; Berghofer et al., 2022; Kwon et al., 2024). The availability of these genomic studies has also enhanced the value of the minipigs as donors for xenotransplantation (Wang Y. et al., 2024; Peng et al., 2025), for toxicological testing (Liu et al., 2008; Bode et al., 2010; Flisikowska et al., 2022), and use as disease models (Curtasu et al., 2019; Curtasu et al., 2020; Li P. et al., 2020; Vaure et al., 2021; Niu et al., 2023; Jia Y. et al., 2024). For example, in the Göttingen minipig, characteristic diet-epigenome interactions were studied for future treatment of obesity, whereas findings in the back muscle of Diannan small-ear pig highlighted the expression of key genes involved in lipid metabolic and fatty acid biosynthetic process, as well as miRNAs regulating lipid deposition and muscle growth (Wang et al., 2015; Feng Y. et al., 2021). Similarly, metabolomic profiling revealed rapid shifts in plasma metabolites after feeding and obesity-related biomarkers after long-term intake of fructose and resistant starch (Polakof et al., 2015; Curtasu et al., 2020). In Bama pigs, promoter-enhancer interactions were shown to be highly dynamic in adipose depots, whereas large-scale compartments of the chromatin and topologically associated domains (TADs) were mostly conserved (Zhang J. et al., 2022).

Building on these insights into tissue-specific growth processes, transcriptome profiling was applied in the pituitary gland to explore the dynamic gene expression patterns during postnatal development in Bama pigs, as well as to characterize mesenchymal stem cell populations in prepubertal Mini-LEWE, providing a broader perspective on growth regulation in minipigs (Shan et al., 2014; Khaveh et al., 2024). Moreover, the establishment of a single-cell transcriptomic profile of mini-pigs has provided a valuable resource for dissecting cell- or nuclei-specific gene expression patterns with applications ranging from postnatal testicular development to metabolic diseases and immune cell maturation (Miao et al., 2024b; Wang X. et al., 2024; Chen et al., 2025; Zhang J. et al., 2025). Subsequently, numerous studies have particularly focused on minipigs for various reasons, including their small size or biomedical relevance.

Nevertheless, there are other vertebrates traditionally raised as mini-livestock, such as members of the family of rodents, e.g., guinea pigs *(Cavia porcellus)*, capybaras (*Cavia aperea*), African mole-rat (*Cryptomys hottentotus*) or cane rat (*Thryonomys swinderianus*), which also underwent omics studies targeting growth, adaptive evolution or their potential as unconventional meat species (Guo et al., 2012; Weyrich et al., 2014; Sahm et al., 2018; Dalle Zotte and Cullere, 2019; Babarinde and Saitou, 2020; Herrera-Alvarez et al., 2021; Schyman et al., 2021; da Silva et al., 2024). Comparative epigenomic profiling suggested that evolutionary changes in regulatory elements underlie key metabolic and physiological adaptations in naked mole-rat and other African mole-rat species, providing insights into their unique traits and offering a new phylogenetic framework for studying regulatory evolution across species (Parey et al., 2023). Omics studies in rabbits *(Oryctolagus cuniculus)* investigated evolutionary dynamics, highlighting gene alleles associated with brain development and aggression related to the domestication process (Albert et al., 2012; Carneiro et al., 2014a; Carneiro et al., 2014b). Alongside tameness, growth and meat quality were highly important traits under selection since domestication of rabbits, which were considered to be an ideal food source with high protein, low fat, low cholesterol and low sodium contents (Yang X. et al., 2020). Thus, marker genes were studied for these desired traits using Specific-Locus Amplified Fragment sequencing (SLAF-seq) and subsequent genome-wide association analysis (Yang X. et al., 2020). Furthermore, various research studies explored the metagenome and metabolome of the rabbit gut, caecum and colon across different conditions, attributed to the impact on host health and adaptation (Chen Y. et al., 2022; Wang J. et al., 2022; Hou et al., 2024; Paes et al., 2024; Zhao et al., 2024).

In addition to mammals, poultry (Table 4) has also become a major focus of omics research aiming at an increased production efficiency, disease resistance and understanding phenomena like epigenetic inheritance (Urgessa and Woldesemayat, 2023; Wadood et al., 2025). Population genomics studies characterized genetic diversity across chicken breeds, detecting selection signatures and identifying candidate genes for economically important traits (Fan et al., 2013; Li D. et al., 2019; Qanbari et al., 2019; Shi et al., 2023; Wang H. et al., 2023; Rabbani et al., 2024). By integrating genomics data with transcriptome information or by detecting differential gene expression levels in chicken, researchers could highlight functional genetic effects affecting egg production performance, feeding efficiency and meat quality (Mutryn et al., 2015; Zhou et al., 2015; Zhuo et al., 2015; Piorkowska et al., 2016; Ye et al., 2020; Huang et al., 2022; Cai et al., 2023; Cui et al., 2024; Liu Y. et al., 2024). Additionally, breast muscle proteomes from chicken kept in antibiotic-free or organic farming systems revealed differentially abundant proteins as putative biomarkers for meat or farming system authenticity (Alessandroni et al., 2024). For future enhanced integration studies of multi-omics chicken data, an omics data repository (GalBase) was constructed to facilitate the identification of genetic variants and functional genes associated with common traits of interest (Fu et al., 2022). Similarly, researchers in the field of duck genomics initiated the Duck 1,000 genomes project, which integrates multi-omics data for studies on economically important traits in ducks (Fan et al., 2024). Particularly, growth, feed conversion efficiency, meat quality and meat yield were investigated intensively using genomics, transcriptomics or mass spectrometry (Liu Y. et al., 2018; He J. et al., 2019; Hu et al., 2021a; Hu et al., 2021b; Cai et al., 2023; Cao et al., 2023; Hu and Liu, 2023; Mohammadi, 2024; Yang Y. et al., 2024) (In contrast, in Muscovy ducks, the strong female behaviour of incubating eggs instead of laying was of major interest and therefore explored with different omics methods (Wu et al., 2019; Lin et al., 2021).

**TABLE 4:** Overview of mini-livestock studies in the field of omics focusing on poultry. The table provides a comprehensive summary of omics studies identified for poultry, detailing the omics category, sequencing methodology, targeted biological features, and overall study design.

Investigated mini-livestock species	Omics field (”-omics”)	Sequencing-technique	Targets and study design	References
Chicken *(Gallus gallus)*	Gen-	WGS	Re-sequencing, selective sweeps, difference between wild and domestic animals	Qanbari et al. (2019)
Chicken *(Gallus gallus)*	Gen-	WGS	Re-sequencing for improving further future investigations	Pertille et al. (2016)
Chicken *(Gallus gallus)*	Gen-	WGS	Re-sequencing of Bangladeshi indigenous chicken, insights into new breeding schemes	Rabbani et al. (2024)
Chicken *(Gallus gallus)*	Gen-	WGS	Genomic footprints and genes influencing body weight in Chinese indigenous chicken, *NELL1, XYLT1*, and *NCAPG/LCORL* strongly selected	Wang et al. (2023a)
Chicken *(Gallus gallus)*	Gen-	WGS	Re-sequencing of chicken from national local poultry genetic resources conservation farm, selection signatures associated with adaptation to tropical and frigid environments	Shi et al. (2023)
Chicken *(Gallus gallus)*	Gen-	WGS	Patterns of variation in two breeds, economic traits, selective sweeps	Fan et al. (2013)
Chicken *(Gallus gallus)*	Gen-	WGS	Patterns of diversity, genome-wide variation, population structure studies, selection signatures	Li et al. (2019a)
Chicken *(Gallus gallus)*	Gen-, Transcript-	WGS, RNA-seq	Candidate genes in egg production of upright and pendulous-comb chicken	Cai et al. (2023)
Chicken *(Gallus gallus)*	Gen-, Transcript-	WGS, RNA-seq	Feed efficiency (residual feed intake), GWAS, reducing environmental impact	Ye et al. (2020)
Chicken (*Gallus gallus*)	Gen-, Transcript-	WGS, RNA-seq	Maternal heat stress, adaptive mechanisms concerning heat tolerance	Karami et al. (2025)
Chicken *(Gallus gallus)*	Transcript-	RNA-seq	Characterization of Wooden Breast phenotype, localized hypoxia, oxidative stress, increased intracellular calcium, and muscle fibre-type switching as key features	Mutryn et al. (2015)
Chicken *(Gallus gallus)*	Transcript-	RNA-seq	Evaluation of the coverage and depth of transcriptome from chicken lungs	Wang et al. (2011)
Chicken *(Gallus gallus)*	Transcript-	RNA-seq	Shear force breast muscles (tenderness), differential gene expression analysis suggests the involvement of the extracellular matrix in the determination of meat tenderness	Piorkowska et al. (2016)
Chicken *(Gallus gallus)*	Transcript-	RNA-seq	Signaling pathways associated with disease resistance to avian influence virus infection	Wang et al. (2014)
Chicken *(Gallus gallus)*	Transcript-	RNA-seq	Gene expression in abdominal fat of high- and low-feed-efficiency in broiler chickens	Zhuo et al. (2015)
Chicken *(Gallus gallus)*	Transcript-	scRNA-seq	Immune cell populations, peripheral blood leukocyte analysis, chicken health	Maxwell et al. (2024)
Chicken *(Gallus gallus)*	Transcript-	RNA-seq	Meat quality characteristics, differential expressed genes, fat metabolism in broiler and laye breeds	Liu et al. (2024c)
Chicken *(Gallus gallus)*	Transcript-	RNA-seq	Insights into feed efficiency, differences between low and high feed efficiency breeds, genes involved in muscle growth	Zhou et al. (2015)
Chicken (*Gallus gallus*)	Transcript-	RNA-seq	Heterosis effects on egg numbers and clutch size, KEGG and REACTOME pathways	Isa et al. (2022)
Chicken (*Gallus gallus*)	Transcript-	RNA-seq	Comparing mRNA and microRNA on oxidative muscle sartorius and glycolytic muscle, candidate genes for muscle fibre type determination	Liu et al. (2020)
Chicken *(Gallus gallus)*	Transcript-, Metabol-	RNA-seq, LC-MS/MS	Egg production mechanisms, analysis of ovaries in two breeds, signalling pathways, genes associated with reproduction	Huang et al. (2022)
Chicken *(Gallus gallus)*	Transcript-, Metbol-	RNA-seq, LC-MS/MS	Integrative analysis, meat quality, myofiber characteristics, different rearing systems	Cui et al. (2024)
Chicken (*Gallus gallus*)	Epigen-	Hi-C	Breast muscle growth and intramuscular fat between fast-growing broilers and slow-growing indigenous chickens, effects of chromatin loops	Wang et al. (2024c)
Chicken *(Gallus gallus)*	Prote-	LC-MS/MS	Protein biomarkers, protein changes in breast meat from divergent farming systems, muscle structure and energy metabolism, muscle proteome	Alessandroni et al. (2024)
Chicken *(Gallus gallus)*	Prote-	MALDI-MS, LC-MS/MS	Qualitative and quantitative approaches, semen peptidome/proteome and molecular phenotype related to sperm quality	Labas et al. (2015)
Duck *(Anas platyrhynchos)*	Gen-	WGS	Developmental wing deformity (angel wings), GWAS	Mohammadi (2024)
Duck *(Anas platyrhynchos)*	Transcript-	RNA-seq	Skeletal muscle growth in two duck breeds, differential gene expression analysis	Cao et al. (2023)
Duck *(Anas platyrhynchos)*	Transcript-	RNA-seq	Transcriptome analysis of Hanzhong Ma duck	Hu et al. (2021b)
Duck *(Anas platyrhynchos)*	Transcript-	RNA-seq	Transcriptome analysis of abdominal fat tissue, circRNAs involved in regulating duck abdominal fat production	Yang et al. (2024b)
Duck *(Anas platyrhynchos)*	Transcript-	RNA-seq	*De novo* assembly, insights into muscle yield and meat quality gene expressions	Liu et al. (2018b)
Duck *(Anas platyrhynchos)*	Transcript-	RNA-seq	Transcriptome analysis of Pekin duck	Hu et al. (2021a)
Duck *(Anas platyrhynchos)* Muscovy Duck *(Cairina moschata)*	Transcript-	RNA-seq	Analysis of hepatic gene expression, *ad libitum* feed options	Herault et al. (2019)
Duck *(Anas platyrhynchos)*	Transcript-, Metabol-	RNA-seq, LC-MS/MS	Skeletal muscle development during embryonic stage, correlation analysis, KEGG-pathway analysis	Hu and Liu (2023)
Duck *(Anas platyrhynchos)*	Prote-	MALDI-TOF MS	Matrix-Assisted Laser Desorption/Ionization Time-of-Flight Mass Spectrometry (MALDI-TOF MS), liver protein profile, functional enrichment analysis, differentially expressed proteins significantly related to lipid metabolic processes	Ge and Geng (2022)
Duck *(Anas platyrhynchos)*	Prote-	TMT, LC-MS/MS	Duck meat from birds exposed to heat stress, potential protective proteins involved in the defensive mechanisms against heat stress	He et al. (2019a)
Goose *(Anser cygnoides)*	Gen-, Transcript-, Epigen-	WGS, RNA-seq, Hi-C	Chromosome-level genome of a Chinese indigenous goose, diversity analysis of two different breeds, insertion in EDNRB2 determines the white plumage	Ouyang et al. (2022)
Goose *(Anser cygnoides)*	Gen-, Epigen-	WGS, Hi-C	Chromosome-level goose assembly generated by adopting a hybrid *de novo* assembly approach	Li et al. (2020b)
Goose *(Anser cygnoides)*	Gen-	WGS	Draft sequence by shotgun sequencing, molecular mechanism involved in the susceptibility of geese to fatty liver disease	Lu et al. (2015)
Goose *(Anser cygnoides)* *(Branta ruficollis*)	Gen-	WGS	Re-sequencing of geese, exon-based phylogenomic approach, evolutionary history, timing of divergence, phylogenetic incongruences	Ottenburghs et al. (2016)
Goose *(Anser cygnoides)* *(Anser anser)*	Gen-	WGS	Population structure analysis of re-sequencing data, introgression of Yili geese by Chinese domestic geese	Wen et al. (2023)
Goose *(Anser cygnoides)*	Transcript-	RNA-seq	Transcriptome of hypothalamus, pituitary, and ovaries, reference for light regulation of reproduction	Zhao et al. (2025a)
Goose *(Anser cygnoides)*	Transcript-	RNA-seq	Transcriptome of hypothalamus of geese under different photocycles	Yuyan et al. (2025)
Goose *(Anser cygnoides)*	Transcript-	RNA-seq	Differentially expressed genes of abdominal fat and breast muscle	Zhao et al. (2025b)
Goose *(Anser cygnoides)*	Transcript-, Prote-	RNA-seq, LC-MS/MS	Reproduction cycles in testicles, genes related to spermatogenesis and fertility	Liu et al. (2024a)
Goose *(Anser cygnoides)*	Transcript-	scRNA-seq	Multi-omics approach with genome sequencing data and single-cell transcriptome data from ovarian tissues from three female embryos, *LHX9* and *ARID5B* as potential regulators of degeneration of the right ovary	Ouyang et al. (2025)
Guinea Fowl – Helmeted *(Numida meleagris)*	Gen-	WGS	Draft assembly, wild and domestic populations, selection signatures related to domestication or importation to Europe, plumage colouration	Vignal et al. (2019)
Guinea Fowl – Helmeted *(Numida meleagris)*	Gen-	WGS	High-quality *de novo* genome assembly, wild and domestic birds, selective signatures in behaviour and locomotion changes	Shen et al. (2021)
Guinea Fowl *(Numida spp.*)	Prote-	nanoLC-MS/MS	Eggshell formation	Le Roy et al. (2019)
Jiaji Duck *(Cairina moschata)*	Transcript-, Metabol-	RNA-seq, LC-MS/MS	Integrated study of embryonic breast muscle, KEGG-pathway correlations and integration, differential gene expression and differential metabolomics analysis	Gu et al. (2024)
Muscovy Duck *(Cairina moschata)*	Prote-, Metabol-	LC-MS/MS	Differentially expressed proteins in ovary tissues during the broody and laying periods	Wu et al. (2019)
Muscovy Duck *(Cairina moschata)*	Gen-, Epigen-, Transcript-	WGS, RNA-seq, Hi-C, ATAC-seq	Multo-omics methods including Assay for Transposase-Accessible Chromatin using sequencing (ATAC-seq), fatty liver, chromosome-level assembly, differential gene expression analysis involved in hepatic lipid catabolism	Xu et al. (2022b)
Muscovy Duck *(Cairina moschata)*	Gen-	WGS	Domestication history, guidance for breeding programs and enhancing commercial traits, genes implicated in fatty acid metabolism, development and immunity pathways	Li et al. (2024a)
Muscovy Duck *(Cairina moschata)*	Transcript-	RNA-seq	Full-length transcriptome from ovaries	Lin et al. (2021)
Quail – Japanese *(Coturnix japonica)*	Gen-	WGS	High-quality reference genome, identification of quail-specific genes, GWAS for plumage colour	Wu et al. (2018)
Quail – Japanese *(Coturnix japonica)*	Gen-	WGS	Draft assembly	Kawahara-Miki et al. (2013)
Quail – Japanese *(Coturnix japonica)*	Transcript-	RNA-seq	Thermal manipulation on eggs impacting postnatal response and gene expression in the hypothalamus, genes related to mitochondrial and heat-response	Vitorino Carvalho et al. (2021)
Quail – Japanese *(Coturnix japonica)*	Transcript-	RNA-seq	Developmental patterns of leg muscles in quail embryos and regulatory networks reconstruction	Zhang et al. (2024b)
Quail – Japanese *(Coturnix japonica)*	Transcript-	RNA-seq	Sexual dimorphism, expression patterns of genes involved in sex-determination mechanisms	Park et al. (2024)
Quail – Japanese *(Coturnix japonica)*	Metabol-	LC-MS/MS	Characterization of the quail liver metabolome, effects on lipid metabolism	Legrand et al. (2021)
Quail – Japanese *(Coturnix japonica)*	Gen-, Transcript-	WGS, RNA-seq	High-quality quail genome sequence, candidate genes for social behaviour, effects and interaction of photoperiod and temperature	Morris et al. (2020)
Quail – Japanese *(Coturnix japonica)*	Gen-	WGS	Copy number variant detection, markers for stress responses	Khatri et al. (2019)
Quail – Japanese *(Coturnix japonica)*	Transcript-	RNA-seq	Skeletal muscle development, regulatory networks	Zhang et al. (2024b)
Quail – Japanese *(Coturnix japonica)*	Transcript-	RNA-seq	Exposure to glucocorticoids, differential gene expression, stressors with long-term effects on the brain	Marasco et al. (2016)
Quail *(Coturnix coturnix)* Duck *(Anas platyrhynchos)* Chicken *(Gallus gallus)*	Prote-	MALDI-TOF/TOF MS	Matrix-Assisted Laser Desorption/Ionization Time-of-Flight/Time-of-Flight Mass Spectrometry (MALDI-TOF/TOF MS), comparison of the protein composition of egg white	Hu et al. (2016)
Turkey *(Meleagris gallopavo)*	Gen-	WGS	Draft assembly, comparative analysis of the turkey, chicken, and zebra finch genomes	Dalloul et al. (2010)
Turkey *(Meleagris gallopavo)*	Gen-, Epigen-	WGS, Hi-C	High-quality chromosome-level assemblies using long-read technologies and genome-wide chromatin interaction data	Barros et al. (2022)
Turkey *(Meleagris gallopavo)*	Gen-	WGS	Re-sequencing of 32 individual turkeys from different populations, genomic regions with low nucleotide variation in domestic turkey	Aslam et al. (2012)
Turkey *(Meleagris gallopavo)*	Gen-	WGS	Re-sequencing, selective sweeps regions, associated with growth	Aslam et al. (2014)
Turkey *(Meleagris gallopavo)*	Transcript-	RNA-seq	Transcriptome profiles of normal, pale and exudative meat	Malila et al. (2014)
Turkey *(Meleagris gallopavo)*	Transcript-	RNA-seq	Liver transcriptome, response to selenium status	Sunde and Taylor (2019)
Turkey *(Meleagris gallopavo)*	Transcript-	RNA-seq	lncRNA regulation associated with sperm motility	Jastrzebski et al. (2022)
Turkey (*Meleagris gallopavo*)	Transcript-	RNA-seq	Identifying and characterizing differential expression of satellite cells from muscles, prediction of miRNA and mRNA interactions, thermal challenge response	Reed et al. (2023)

Similarly to the duck’s reference genome, research benefited significantly from the availability of a chromosome-level assembly from Tianfu goose *(Anser cygnoides*) or a hybrid *de novo* assembly from Chinese indigenous goose, as well as the recent introduction of a “Goose Multi-omics Database” (Li Y. et al., 2020; Ouyang et al., 2022; Huang et al., 2025). Subsequent re-sequencing allowed studies on the evolutionary history of geese and gene flow in domestic populations (Ottenburghs et al., 2016; Wen et al., 2023). Furthermore, transcriptomic approaches using RNA-sequencing elucidated molecular mechanisms underlying economically important traits, including the identification of key genes for differential fat deposition, photoperiodic reproductive control and fertility (Liu Q. et al., 2024; Yuyan et al., 2025; Zhao X. et al., 2025). In a multi-omics joint analysis, the integration of genomic variants and single-cell transcriptomic information allowed the detection of two genes, *LHX9* and *ARID5B*, potentially associated with the rate of degeneration in the right ovary of avian species (Ouyang et al., 2025).

Significant progress has also been made in the turkey *(Meleagris gallopavo)*, where an initial draft genome was improved to a high quality, chromosome-level assembly (Dalloul et al., 2010; Barros et al., 2022). Subsequent population analyses identified large genomic regions under intense selection in commercial lines enriched for economically relevant genes (Aslam et al., 2012; Aslam et al., 2014). Furthermore, transcriptome sequencing studies provided insights into sperm motility, meat quality parameters and adaptation to high dietary selenium (Malila et al., 2014; Sunde and Taylor, 2019; Jastrzebski et al., 2022).

Comparable advances have also been made in the Japanese quail (*Coturnix japonica*), for which recent genome, transcriptome and mass spectrometry data provided valuable insights into egg white protein functions, lipid metabolism, social behaviour, stress responses, effects of photoperiod and temperature on eggs and birds, muscle development and plumage colour (Hu et al., 2016; Marasco et al., 2016; Wu et al., 2018; Khatri et al., 2019; Morris et al., 2020; Legrand et al., 2021; Vitorino Carvalho et al., 2021; Zhang W. et al., 2024). Similar traits were also of interest in studies on helmeted guinea fowl *(Numida meleagris),* in which signatures of selection in the genome were investigated for behaviour and locomotion changes or plumage colouration (Vignal et al., 2019; Shen et al., 2021).

Another group of vertebrates, namely amphibians and reptiles (Table 5), are also utilized as edible animals, providing alternative sources of high-quality protein in various regions of the world, and has increasingly been studied using omics approaches. For example, frogs are a popular source of meat in aquaculture worldwide and, like conventional livestock, have implications for both human nutrition and health (Boss et al., 2023; Zhang L. et al., 2025). Genomic studies in different frog species highlighted multi-genome synteny blocks, mechanisms driving or constraining genome size and positively selected genes for adaptation to high altitude (Sun et al., 2015; Lamichhaney et al., 2021; Chen et al., 2023). Transcriptome analyses in the American bullfrog *(Lithobates catesbeianus)* and the Heilongjiang brown frog *(Rana amurensis)* revealed target pathways for metabolism and immune response, as well as antimicrobial peptides in the skin (Li W. et al., 2022; Yang P. et al., 2024). In addition, chromosome-level assemblies and *de novo* transcriptome datasets of reptiles, such as edible lizards and salamanders, have been generated, providing valuable resources for studying their physiology and adaptation (Geng et al., 2017; Yurchenko et al., 2020; Westfall et al., 2021; Wang Q. et al., 2023). In summary, the application of omics technologies in a wide range of vertebrate mini-livestock has provided comprehensive insights into their genetic architecture, physiological regulation, and adaptive diversity.

**TABLE 5:** Overview of mini-livestock studies in the field of omics focusing on amphibians and reptiles. The table provides a comprehensive summary of omics studies identified for amphibians and reptiles, detailing the omics category, sequencing methodology, targeted biological features, and overall study design.

Investigated mini-livestock species	Omics field (”-omics”)	Sequencing-technique	Targets and study design	References
Bullfrog – American *(Lithobates catesbeianus)*	Transcript-	RNA-seq	Gene expression profiles in the liver, significant pathways in metabolism and immune response	Yang et al. (2024a)
Bullfrog – American *(Rana catesbeiana)*	Transcript-	RNA-seq	Functional investigation of the prestin (SLC26A5) gene and its evolution	Wang et al. (2021b)
Frog – Chinese Edible Frog *(Hoplobatrachus rugulosus)*	Gen-	WGS	Resistance to clinical antimicrobials, multidrug resistance, *Salmonella* as agent of gastrointestinal disease, high number of virulence determinants	Boss et al. (2023)
Frog – Giant Spiny Frog *(Quasipaa spinosa)*	Gen-	WGS	Genome re-sequencing, sex specific markers	Zhang et al. (2025b)
Frog – Heilongjiang Brown Frog *(Rana amurensis)*	Transcript-	RNA-seq	Comparative studies, samples from different organs, antimicrobial peptides in the skin	Li et al. (2022c)
Frog – Ornate Burrowing Frog *(Platyplectrum ornatum)*	Gen-, Transcript-	WGS, RNA-seq	Mechanisms of genome reduction in comparison to other frog species, sex-differentiation pathways	Lamichhaney et al. (2021)
Frog – Plateau Brown Frog *(Rana kukunoris)*	Gen-	WGS	Chromosome-level assembly, amphibian genome evolution and adaptation	Chen et al. (2023)
Frog – Tibetan *(Nanorana parkeri)*	Gen-, Transcript-	WGS, RNA-seq	Synteny analysis, transposable elements	Sun et al. (2015)
Lizard – Common *(Zootoca vivipara)*	Gen-	WGS	Chromosome-level assembly, high-density linkage map, novel analytic pipelines	Yurchenko et al. (2020)
Lizard – Easter Fence Lizard *(Sceloporus undulatus)*	Gen-, Transcript-	WGS, RNA-seq	Chromosome-level assembly, physiological and evolutionary ecology	Westfall et al. (2021)
Lizard – Oriental Garden Lizard *(Calotes versicolor)*	Gen-. Transcript-	WGS, RNA-seq	Draft genome, genome landscape, phylogenetic relationship	Wang et al. (2023b)
Salamander – Chinese Giant Salamander *(Andrias davidianus)*	Transcript-	RNA-seq	*De novo* transcriptome assembly	Geng et al. (2017)

## 6 Translating omics insights into mini-livestock improvement: challenges and future directions

The rapid advancement of omics technologies has generated extensive datasets describing the genetic, transcriptomic, proteomic, and metabolic architecture of mini-livestock. Harnessing these insights into practical breeding applications enables more accurate selection for desirable traits, such as growth, meat quality, fat metabolism, feed intake, tameness, and other key productive traits (Albert et al., 2012; Jegou et al., 2016; Southey et al., 2016; Zhong et al., 2022; Zhao Z. et al., 2025).

The global livestock sector faces the dual challenge of ensuring food security for a growing population while controlling its environmental footprint (Niu et al., 2024). In livestock science, omics technologies have become powerful tools for unravelling the molecular mechanisms underlying complex traits, thereby supporting more sustainable and efficient animal production (Chakraborty et al., 2022). Genomics accelerate the early selection of traits of interest, such as milk yield for dairy cows or the quantity of meat produced by beef cattle (Mesbah-Uddin et al., 2022; Nanaie et al., 2025). While these approaches have already advanced genetic improvement in major livestock species, their application in mini-livestock remains comparatively limited. However, the availability of high-quality reference genomes and transcriptomic datasets has recently enabled the first insights into the molecular backgrounds of important production and adaptation traits in these species (Chen et al., 2012; Generalovic et al., 2021; Ouyang et al., 2022; Wy et al., 2024; Chen et al., 2025). Sequencing or re-sequencing of mini-livestock species such as chicken, rabbit or honey bee resulted in millions of SNPs to identify genotype to phenotype correlations for complex traits (Carneiro et al., 2014b; Wallberg et al., 2017; Moreira et al., 2018).

While these omics applications yielded valuable insights into mini-livestock biology and production, several challenges and limitations remain to be addressed before their full potential can be realized in mini-livestock breeding and management. Omics technologies represent a paradigm shift in the optimization of mini-livestock systems by enhancing productivity, sustainability and biomedical relevance as already demonstrated in agricultural and conventional livestock species (Van Emon, 2016; Chakraborty et al., 2022; Rotimi, 2025). A primary limitation is the requirement for highly equipped laboratories and expensive instrumentation for omics data generation (Van Emon, 2016; Chakraborty et al., 2022). Protocols for sample collection and processing have to be optimized for each species and tissue type, as for example in insects, in which the pupal stage was suggested as the most suitable developmental stage for high-quality genomic DNA extraction (Oppert et al., 2019). Downstream of sequencing, the assembly of high-quality reference genomes for many species, particularly insects, remains challenging due to high polymorphism, limited DNA yield from small-bodied individuals, and difficulties in achieving homozygosity (Richards and Murali, 2015; Li F. et al., 2019). Data analysis on omics research requires substantial expertise in statistics, bioinformatics, and computational tools to ensure accurate interpretation (Yamada et al., 2021). Analytical frameworks such as statistical models and network-based approaches evolve rapidly, demanding continual updates to maintain performance (Jiang et al., 2019). Beyond the challenges of handling large and complex single-omics datasets, data integration remains a major obstacle for effective multi-omics analyses (Misra et al., 2019). Furthermore, the implementation of omics technologies in livestock faces practical challenges, as farmers’ acceptance largely depends on the extent to which these tools align with their expertise and operational needs (Hostiou et al., 2017; Vazquez-Diosdado et al., 2019). Thus, handling omics data requires domain-specific knowledge and proper study design to harness the full potential, particularly considering the growing scale of datasets and the economic importance of traditional livestock species (Thornton, 2010).

These limitations discussed above underline the need for future strategies that scale from individual studies to coordinated, community-driven efforts. Experiences from large international consortia demonstrate that shared standards, common data models, and openly accessible workflows are crucial for reproducibility and for lowering the entry barrier into omics research: The initiative of the Functional Annotation of Animal Genomes (FAANG) project, whose aim is to produce comprehensive maps of functional elements in the genomes of domesticated animal species, has already shown significant progress in filling the genotype-to-phenotype gap and the establishment of data analysis standards for various species, including mini-livestock (Andersson et al., 2015; Tuggle et al., 2016). Similarly, the ENCODE Consortium provides clear examples of how community-curated benchmark datasets, guides and uniform data-processing methods can provide comprehensive views of the organization and variability of genes and regulatory information across species and individuals (ENCODE Project Consortium, 2012). Furthermore, the 5,000 arthropod genomes initiative (i5K) highlighted that such coordination was also feasible for less conventional livestock and agricultural species (i5K Consortium, 2013). The European Reference Genome Atlas (ERGA) model, which distributed sequencing across 26 facilities and established coordinated library preparation hubs, offers valuable lessons for scaling mini-livestock genomics programs while ensuring broader participation from researchers in resource-limited settings (Mc Cartney et al., 2024).

A further major development in this context is the implementation of pangenome frameworks representing the global genomic diversity of a species rather than a singular reference (Wang T. et al., 2022). Such approaches are now beginning to appear also in non-traditional model organisms, for example, in the Asian honeybee *(Apis cerana)*, in which the first pangenome recently demonstrated its feasibility in the analysis of genomic variations (Li Y. et al., 2024). This highlights the potential of transferring existing pangenome-related strategies from traditional livestock to mini-livestock to identify variants involved in phenotypic and genotypic diversity, environmental adaptation mechanisms and other desired phenotypes (Zhou et al., 2022; Miao et al., 2024a; Azam et al., 2025; Dai et al., 2025). Beyond improving variant discovery, these resources also provide a foundation for developing cost-effective genotyping approaches. Established procedures in traditional livestock, such as low-density SNP panels combined with imputation (van Binsbergen et al., 2015), genotyping-by-sequencing (Elshire et al., 2011), and low-coverage whole-genome imputation (Zhang et al., 2023), demonstrated that accurate genomic information could be obtained to perform large-scale genome-wide association studies and genomic selection. As more comprehensive variant catalogues and pangenomes become available, these methods could also be tailored to species and breeds where no commercial genotyping tools currently exist (Crysnanto et al., 2019; Bian et al., 2024).

Addressing the remaining gaps in omics applications will additionally require integrating phenotype-level information and advancing multi-layer analytical strategies (Brito et al., 2020). Artificial intelligence-driven phenotyping tools offer a promising route toward generating standardized, high-throughput behavioural and performance data, which are essential for linking molecular variation to observable traits (Distante et al., 2025). Developing applications in insect farming already demonstrated that artificial intelligence can automate identification, counting, behaviour detection, and health monitoring, providing scalable solutions for mini-livestock systems where manual data collection is difficult (Bjerge et al., 2022; Roy et al., 2024). When combined with genomic and management strategies, these technologies have the potential to improve production efficiency and reduce costs (Neculai-Valeanu et al., 2025; Papadopoulos et al., 2025), which are the key factors for ensuring the long-term sustainability and competitiveness of mini-livestock production systems in the future.

## 7 Conclusion

Omics technologies have demonstrated transformative potential across diverse mini-livestock species, as outlined in this review. Integrating multi-omics approaches has greatly enhanced our understanding of genetic architecture, physiological regulation, and adaptive diversity. Future advances will rely on expanding reference genomes, standardizing phenotyping, and applying robust integration frameworks, such as CCA, WGCNA, pathway-based enrichment tools, and machine-learning approaches. Together, these strategies can accelerate trait mapping and support breeding programs targeting growth efficiency, food and feed utilization, product quality, and resilience to environmental stressors. From a policy perspective, embedding multi-omics integration into agricultural strategies can facilitate evidence-based breeding, emission-reduction goals, and internationally harmonized data-sharing standards.
